# Development and psychometric evaluation of a new tool for measuring the attitudes of patients with progressive neurological diseases to ethical aspects of end-of-life care

**DOI:** 10.1186/s12910-020-00471-9

**Published:** 2020-04-15

**Authors:** Radka Bužgová, Radka Kozáková

**Affiliations:** Department of Nursing and Midwifery, Faculty of medicine, University of Ostrava, Ostrava, Czech Republic

**Keywords:** Attitudes, Care, End of life, Neurology, Reliability, Validity

## Abstract

**Background:**

Knowing the opinions of patients with Progressive Neurological Diseases (PNDs) and their family members on end-of-life care can help initiate communication and the drawing up of a care plan. The aim of this paper is to describe the creation and psychometric properties of the newly developed APND-EoLC questionnaire (the *Attitudes of Patients with Progressive Neurological Disease to End of Life Care* questionnaire).

**Methods:**

Following focus group discussion, four main areas of interest were identified: patients’ and family members’ attitudes towards end-of-life care, factors influencing decisions about treatment to prolong patients’ life, concerns and fears regarding dying, and opinions on the system of care. The created questions were divided into domains based on factor analysis and psychometric properties were evaluated by sample of 209 patients with PND and 118 their family members.

**Results:**

The final version of the scale contains a total of 28 questions divided into six domains (end-of-life control, keeping patients alive, trust in doctors/treatment, trust in social support, sense of suffering, and dependence/loss of control) and five individual questions determining views of the care system with specified response options. Construct validity was verified by confirmatory factor analysis for each evaluated area individually. Appropriate psychometric properties were identified in the questionnaire.

**Conclusions:**

The APND-EoLC questionnaire can be recommended for use in both research and clinical practice.

## Background

Over the past 10 years, there has been increasing professional interest in the provision of quality end-of-life care in patients with progressive neurological diseases (PND), and studies regarding the implementation of palliative care in neurology [1–10], or more specifically in the care of patients with Parkinson’s disease (PD) [11–13], motor neuron disease (MND) [14, 15], and multiple sclerosis (MS) [16–18], have been published.

In 2008, The European Association for Palliative Care, together with the European Academy of Neurology (EAN) initiated the first discussions about the development of palliative care in patients with neurological disease with the aim of improving cooperation between palliative and neurological care providers, and thus to improve care for persons with advanced PND [4].

PND, such as PD, MS, amyotrophic lateral sclerosis (ALS), MND, or Huntington’s disease (HD) are characterized by a combination of movement disorders, cognitive function disorders, emotional, and behavioral disorders with varying rapid progression [6]. They can also affect individuals of productive age. They are a diverse group of patients with varying dynamics of gradual deterioration.

As part of the AZV MZ ČR project “Neuropalliative and Rehabilitation Care in Patients with Progressive Neurological Disease”, focus groups were organized with the aim of integrating this model into the current system of provision of health and social care to these patients [19]. Since patients, family members, and professionals have different views of how patients with PND and their family members perceive end-of-life care (EoLC), we decided to create a questionnaire identifying the ethical aspects of end-of-life care, including opinions and attitudes of patients with PND and their family members to healthcare provision in the advanced stages of the disease. After a review of the literature, we discovered that there are few available measuring tools to determine PND patients’ views on and attitudes to end-of-life care.

A large proportion of end-of-life care assessment tools focus on assessing attitudes to care/ treatment (pain management, end-of-life decision-making, previously expressed wishes, euthanasia, assisted suicide, end-of-life nutrition, and communication) from the doctors’ perspective [20], reflecting doctors’ attitudes to end-of-life law [21], and doctors’ attitudes toward hastened death [22] or palliative sedation [23, 24]. In addition, there are studies focusing on assessment of nursing students’ attitudes to end-of-life care [25–27], in which students’ attitudes are identified using the *Frommelt Attitudes towards Care of the Dying Part B Scale”,* a 30-item scale (15 positive and 15 negative attitudes) in which respondents agree/disagree with each attitude on a five-point scale.

Clarke et al. [28] investigated preferences of the public to end-of life issues in cases of loss of cognitive/decision-making abilities in Great Britain and the USA. The authors created their own questionnaire in which they determined public opinion of maintaining life at any cost versus assistance in peaceful dying in five stages of disease (dementia) progression. In 2005, Catt et al. [29] created the 27-item Attitudes of Older People to End-of-Life Issues (AEOLI). The AEOLI questionnaire assesses attitudes of older people to death, palliative treatment, and hospice care [29] on a five-point Likert Scale, ranging from “strongly agree” to “strongly disagree” in the following areas: decision making, pain, care environment, living wills, euthanasia/physician-assisted suicide, ageism, psychological needs including religious/spiritual, quality versus quantity of life, and societal awareness. Park et al. [30] investigated the attitudes of older people to death and do-not-resuscitate orders in Korea. Hunt et al. [31] investigated the experiences of end-of life care in the elderly and their preferred places of dying.

Only one study was found that evaluated opinions of patients with PND on end-of-life care, for patients with ALS. Ganzini et al. [32] determined ALS patients’ attitudes to assisted suicide. Patients and their family members agreed or disagreed with statements regarding the following areas: refusal of life-saving medical treatment (including cardiopulmonary resuscitation, mechanical ventilation, feeding tubes, and use of adequate medication for pain) even if death was hastened as a result; attitudes to euthanasia; and attitudes to assisted suicide. The created questionnaire was not validated.

The aim of this paper is to describe the development and psychometric properties of a newly created questionnaire for the evaluation of views on and attitudes to ethical aspects of end-of-life care.

## Methods

### Questionnaire development

**Fig. 1:**
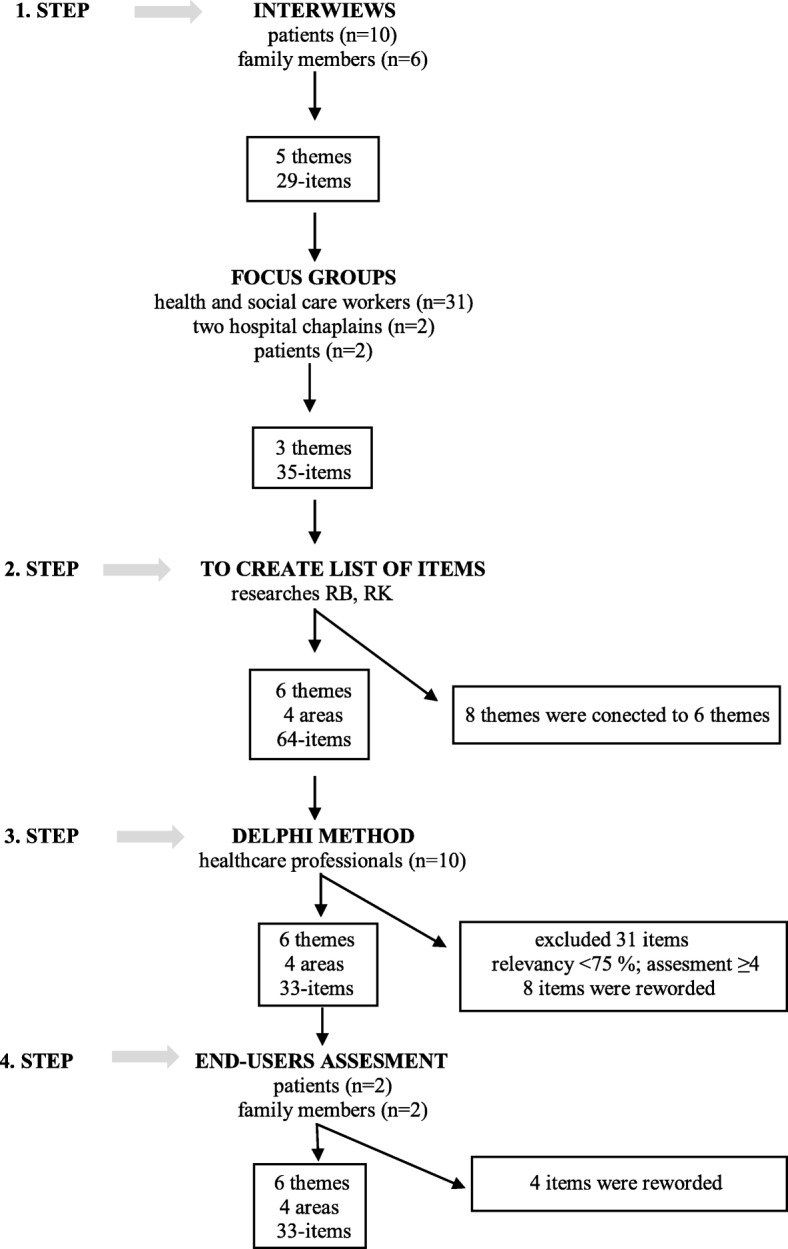
Phases of questionnaire development

The APND-EoLC (*The Attitudes of Patients with Progressive Neurological Disease to End-of- Life Care Questionnaire*) was created in four individual phases (see Fig. 1):

1. The purpose of first phase was identified a problematic issues of end of life care for domains and items generation. The first phase included a individual in-depth interviews with PND’s patients (four with severe MS, six with severe PS), family members of PND’s patients (two with MS, one with PD, one with HD, two with MND) and a discussion in focus groups (*n* = 4), consisting of 31 health and social care workers (doctors, nurses, physiotherapists, occupational therapists, psychologists, and social workers), two hospital chaplains, and two patients – one with MS, one with PD). The selection of the participants was intentional, and based on the stated criteria: 1) a patient, or family member of a patient with a specific neurological disease (multiple sclerosis, Parkinson’s disease, atypical parkinsonism, Huntington’s disease, motor neuron disease), at least 1 year after the diagnosis was made, age > 18 years, MMSE ≥24 points, informed consent in writing; 2) professionals – professional qualification for the given position, at least 1 year’s experience of providing care to patients with neurological disease. The data collection formed part of a qualitative study for the research project run by the Czech Ministry of Health (no. 17–29,447) entitled “Neuro-palliative and Rehabilitation Approach to Maintaining Quality of Life of Patients in Advanced Stages of Specific Neurological Diseases”. The participants were contacted in all regions of the Czech Republic. The method of obtaining the sample was the snow ball technique. The experiences of the participants with the specific topic was emphasized.

The topic of discussion was the introduction of the concept of neuropalliative and rehabilitative care in patients with PNDs in the Czech Republic, including end-of-life care in patients with PND.

First, individual interviews were conducted with patients (*n* = 10) and family members (*n* = 6). The reason for the individual interviews was the preference of patients and family members for the individual conversation, not participation in the focus group. The individual interviews lasted 30 to 70 min. Problematic ethical issues of the end of life (decision making, dying, fear, information, end of life control) identified by patients and family members in individual interviews were categorized into 35 items and subsequently submitted to focus groups. Participants of focus groups talked about the topics presented and categorized items and subsequently identified other potential topics (a place of end of life care, trust, awareness) and items (*n* = 29). The length of the focus group was 120 min.

All interviews and focus groups were recorded on a voice recorder and then literally transcribed. The acquired data were then organized and described in detail through thematic analysis. The thematic analysis is based on moving back and forth among the individual data segments, among the extracts, and data codes, and their analysis. The analysis was performed in five phases [33]: 1. acquainting with the data - two researchers (RB, RK) independently codified the raw transcript; 2. generating initial codes; 3. theme search - analysis of codes and data to suggest wider meaning patterns (potential themes - end-of-life treatment and care, decision-making and influencing factors, fear of dying); 4. elaborating the topics and their revision; 5.defining and naming the themes (four themes; 64 sub-themes).

2. In the second phase, the questionnaire itself was created. The purpose of the second phase was to obtain a list of possible items to include in the instrument. Based on focus group analysis, two researchers (RB and RK) anticipated problematic end-of-life care issues (end-of-life treatment and care, decision-making and influencing factors, fear of dying), identified areas, and designed individual areas and items (four areas, 6 themes, 45 individual items). Six themes were potential domains.
3. In the final phase, the Delphi method was used to determine expert consensus. The questionnaire was distributed to other selected healthcare professionals from clinical practice (*n* = 10), who expressed their views on the included questions. This expert panel of professionals was formed according to the following criteria: the minimum of 3 years experience with PND’s patients, working with neurology patients in health or social institutions in Czech republic. This ensured content validity of the scale.

Selected experts were sent a questionnaire with individual items whose relevance for assessing attitudes to ethical aspects of end-of-life care was established on a 5-point scale: 1 (the least relevant) – 5 (the most relevant) and one open question for comments. A final version of the questionnaire included items with an expert agreement of at least 75% (rating ≥ 4). In total, 33 items were included in the final version of the questionnaire (28 scale items on a ten-point scale and five items with a specified response option). Now, each comment was reviewed for this pertinence by researchers RK and RB and through consensus was reworded when judged appropriate (8 items).

4. Next, researchers RB and RK created the final version of the questionnaire, containing the most frequently preferred questions grouped in four selected areas. Items were further refined based on feedback from experts. The tool was then handed out to the original two patients and two family members for final comments, which were then considered by the authors before making final revisions. The purpose of this last step was to ensure the face validity of the final set of items with the target population of end-users. Face validity is the “degree that end users judge that the items of an assessment instrument are appropriate to the targeted construct and assessment objectives [34]. Selected patients and family members were interviewed on the appropriate construct and understanding and clarity of the items. Four items were reworded.

Then, the questionnaire was then administered to selected family members and PND patients to test its psychometric properties (see the sample). The final version of The APND-EoLC (*The Attitudes of Patients with Progressive Neurological Disease to End-of- Life Care Questionnaire*) is in additional file 1.

### Psychometric evaluation

#### Sample

Patients and family members meeting the following criteria were included in the research: a patient or a family member of a patient with selected PND (MS, PD, atypical Parkinsonism, HD), at a minimum of 6 months after diagnosis, age > 18 years, and Mini-Mental State Examination MMSE ≥24 points. The total sample consisted of 327 participants (209 patients with PNDs, and 118 family members).

The study conformed to the provisions of the Declaration of Helsinki, and was approved by the ethics committees of University Hospital Ostrava (no. 486/2016). All subjects gave their informed consent to inclusion before they participated in the study.

#### Data analysis

The data were processed using the SPSS v. 21 program. For evaluation of the psychometric properties of the questionnaire, an item analysis of the individual scales was performed, their internal consistency (Cronbach’s alpha) and the correlation between the scales (Spearman’s correlation coefficient), and items within the scales were determined. To verify construct validity, exploratory factor analysis by principal component method with Varimax rotation, was used. Prior to factor analysis, the suitability of this procedure was verified using the Kaiser-Meyer-Olkin (KMO) test, and Bartlett’s test of sphericity. The criteria used to find the best fitting structure and the right number of factors were Eigenvalues greater than 1.0, Cattell’s Scree Test, a factor loading cut-off of 0.30, the percentage of total variance explained, and the plausibility of the factorial solution.

Internal consistency was determined using Cronbach’s alpha (α). While α > 0.70 is usually stated as an acceptable minimum [35, 36], Streiner and Norman [37] state an acceptable minimum value of α = 0.65–0.70. This criterion was also used in our research. We also evaluated Cronbach’s alpha for the domain without an item. If it was higher than a domain score, the item could be reassigned to another domain. Additionally, item-total correlation was performed. We considered the value r > 0.40 to be an acceptable minimum [38]. For all analyses, two-tailed *p* values < 0.05 were considered significant.

## Results

### APND-EoLC questionnaire

For the final version of the APND-EoLC (*The Attitudes of Patients with Progressive Neurological Disease to End-of-Life Care questionnaire*) 28 scale questions (items) were selected to establish participants’ views and attitudes on a ten-point scale, and for five items participants could choose from specified response options. The questions were divided into four individual areas:

I. A scale determining patients’ and family members’ attitudes to end-of-life care (end-of-life control, keeping a patient alive, trust in doctors) - 12 items with a ten-point scale: 1 (totally disagree) - 10 (absolutely agree).
II. A scale determining factors influencing decision-making regarding life-saving treatment - seven items with a ten-point scale: 1 (not at all) – 10 (to a great extent).
III. A scale determining concerns and fear of dying - nine items with a ten-point scale: 1 (not at all) – 10 (to a great extent).
IV. Questions determining views of the care system – awareness, persons making decisions about end-of-life care, and the place of care provision (five questions with specified response options).

The questionnaire was evaluated in separate individual areas. In areas 1–3, domain scores were evaluated. The questions were divided into domains based on factor analysis. The domain score was calculated as the sum of answers to all questions in the domain divided by the number of questions in the domain, and then converted to a 10–100 scale. In area four, individual questions were evaluated separately. Versions of the questionnaire for patients (APND-EoLC_p) and for family members (APND-EoLC_fam) were created. The purpose of the family questionnaire was to assess their own attitudes in this area. Using similarly formulated questions for patients and their family members, questionnaires can be used to compare attitudes to ethical aspects of end-of-life care in patients and their families. This may open a subsequent discussion on these topics when communicating with the patient and their family.

### Socio-demographic characteristics of the sample

A total of 209 patients with PND (MS: *n* = 133, PD: *n* = 69, HD: *n* = 7) and 118 family members (MS: *n* = 40, PD: *n* = 65, HD: *n* = 12) participated in the research. The patients’ average age was 55.1 years (s = 16.4; min-max: 19–91). The family members’ average age was 54.5 years (s = 14.8; min-max: 21–84). A total of 94 family members of patients also completed the questionnaire. Other socio-demographic factors are given in the Table 1.

**Table 1:** Socio-demographic Characteristics of the Sample

	Patients	Family		Patients	Family
	*N* = 209	*N* = 118		*N* = 209	*N* = 118
**Sex –** N (%)			**Job** – N (%)		
Man	74 (35)	53 (45)	Employed	54 (26)	60 (51)
Woman	135 (65)	65 (55)	Student	2 (1)	2 (2)
**Education** N (%)			On maternity leave/at home	3 (1)	5 (4)
Elementary	13 (6)	7 (6)	Invalid pensioner	78 (38)	7 (6)
Secondary	162 (78)	79 (67)	Old-age pensioner	69 (33)	43 (36)
Tertiary	34 (16)	32 (27)	Unemployed	3 (1)	1 (1)
**Children –** yes N (%)	175 (84)	94 (80)	**Duration of disease** – N (%)		
**Marital status –** N (%)			Less than a year	8 (4)	–
Single	32 (15)	18 (15)	1–3 yrs	27 (13)	–
Married	125 (60)	83 (70)	4–6 yrs	40 (19)	–
Divorced	31 (15)	16 (14)	7–10 yrs	29 (14)	–
Widow/er	21 (10)	1 (1)	More than 10 yrs	105 (50)	–
**Relationship** N (%)			**Contribution to care -** N (%)		
Husband/wife	–	58 (49)	None	114 (55)	–
Partner	–	8 (7)	I	25 (12)	–
Son/daughter	–	29 (25)	II	25 (12)	–
Mother/father	–	8 (7)	III	36 (17)	–
Other	–	15 (12)	IV	9 (4)	–

### Evaluation of construct validity and reliability

Construct validity was verified by exploratory factor analysis for each evaluated area individually. Factor analysis was always performed for a group of patients and family members.

#### Area I.: scales identifying attitudes to end-of-life care

Satisfactory values of KMO = 0.771 in the sample of patients, and KMO = 0.709 in the sample of family members were found, meaning use of factor analysis was valid. The suitability of the use of factor analysis was also confirmed by Bartlett’s test of sphericity (patient sample: chí^2^ = 655.670; Df = 66; *p* < 0.001; family member sample: chí^2^ = 573.925; Df = 66; *p* < 0.001). Additionally, we determined communality after factor extraction. Variability of variables is explained by factor analysis from 21 to 26%. Factor analysis included factors whose dispersion was greater than 1. All items of two factors loaded above 0.30.

Exploratory factor analysis of all items in this area divided answers into two different factors (Table 2) in the sample of patients and family members. The first factor, which explains 27% variation (25% in the sample of family members), was called *“control over end-of life”*. It focused on attitudes related to the possibility of influencing the end of life, with an emphasis on its quality rather than length. The second factor, explaining 21% of variation, was called *“keeping patients alive”,* and included attitudes related to saving life and the emphasis put on a doctor taking responsibility for the management of end-of-life care. For the “control over end-of-life” scale, Cronbach’s alpha = 0.652 (respectively 0.659 – sample of family members); while for the “keeping patients alive” scale, Cronbach’s alpha = 0.658 (respectively 0.695 – sample of family members).

**Table 2:** Exploratory Factor Analysis of Items in Area 1: “Attitudes to end-of-life care”

Rotated Component Matrix^a^						
Attitudes	Patients			Family members		
	1	2	α^b^ α^c^	1	2	α^b^ α^c^
**Dom 1 – CONTROL OVER END OF LIFE**			0.652			0.659
Having medicine available to end one’s life.		**0.648**	0.537		**0.554**	0.471
Quality of life is more important than its length.		**0.673**	0.531		**0.497**	0.551
Greater fear of helplessness and dependence than of death.		**0.752**	0.479		**0.714**	0.500
Having pain killers under control.	0.412	**0.476**	0.557		**0.619**	0.552
More open discussions in public about death and dying.	0.340	**0.346**	0.609		**0.309**	0.622
**Dom 2 – KEEPING PATIENTS ALIVE**			0.658			0.695
Discussion with a doctor about prognosis and end of life is too depressing.	**0.309**		0.671	**0.461**		0.645
To have the latest treatment available regardless of the side effects.	**0.537**		0.595	**0.624**		0.587
The doctor should decide about and manage end-of-life care.	**0.427**		0.622	**0.323**	0.316	0.612
To have pain relief treatment at the cost of sedation or confusion.	**0.416**		0.644	**0.529**		0.650
When losing the ability to eat, to start tube (enteral) feeding.	**0.792**		0.652	**0.672**		0.557
When losing the ability to breathe, to introduce APV.	**0.738**	−0.397	0.518	**0.762**		0.528
To be kept alive at any cost.	**0.509**	− 0.499	0.587	**0.638**	−0.323	0.596
	26.8%	21.4%		25.4%	21.1%	

^a^Varimax with Kaiser Normalization, Coefficients below 0.3 suppressed, ^b^Cronbach’s alpha for domain, ^c^Cronbach’s alpha if item deleted

#### Area II: scales identifying factors that influence decision-making

Satisfactory values of KMO = 0.782 in the patient sample, and KMO = 0.714 in the family member sample were found, meaning use of factor analysis was valid. The suitability of the use of factor analysis was further confirmed by Bartlett’s test of sphericity (patient sample: chí^2^ = 664.932; Df = 21; *p* < 0.001; family member sample: chí^2^ = 283.629; Df = 21; *p* < 0.001). Variability of variables is explained by factor analysis from 16 to 52%.

Based on factor analysis, the items were divided into two factors (domains): “trust in the doctor/treatment”, and “trust in social support”. Reliability of the scale was found to be satisfactory, i.e. α > 0.65 (Table 3).

**Table 3:** Exploratory Factor Analysis of Items in Area II: “Factors influencing decision-making”

Rotated Component Matrix^a^						
Attitudes	Patients			Family members		
	1	2	α^b^ α^c^	1	2	α^b^ α^c^
**Dom 3 TRUST IN THE DOCTOR/TREATMENT**			0.852			0.804
vDoctor’s recommendation	**0.872**		0.815	**0.833**		0.734
More doctors’ consensus	**0.901**		0.828	**0.869**		0.760
Hope of a better quality of life	**0.643**	0.536	0.801	**0.7795**		0.722
Hope of prolonging life	**0.622**	0.322	0.801	**0.617**	0.381	0.804
**Dom 4 TRUST IN SOCIAL SUPPORT**			0.652			0.679
The approval of the closest family		**0.358**	0.584		**0.608**	0.592
Information from mass media and the Internet		**0.822**	0.532		**0.835**	0.621
Other patients’ experiences	0.376	**0.740**	0.399	0.399	**0.637**	0.507
	52.1%	15.9%		45.4%	17.8%	

^a^Varimax with Kaiser Normalization, Coefficients below 0.3 suppressed, ^b^Cronbach’s alpha for domain, ^c^Cronbach’s alpha if item deleted

#### Area III: “Scales Identifying Fear of Dying”

In the third area, the suitability of the use of factor analysis was also confirmed (patients: KMO = 0.861; chí^2^ = 841.136; Df = 36; *p* < 0.001; family members: KMO = 0,835; chí^2^ = 525.960; Df = 21; *p* < 0.001). Variability of variables is explained by factor analysis from 13 to 51%. The first factor explained 51% variation in both samples and it combines items ascertaining fear and concerns about one’s own suffering (see Table 4). Reliability of the scales in this area was also found to be satisfactory.

**Table 4:** Exploratory Factor Analysis of Items in Area III: “Fear of dying”

Rotated Component Matrix^a^						
Attitudes	Patients			Family members		
	1	2	α^b^ α^c^	1	2	α^b^ α^c^
**Dom 5 - ONE’S OWN SUFFERING**			0.828			0.822
Severe pain	**0.702**		0.812	**0.618**		0.818
Loneliness	**0.780**		0.799	**0.796**		0.765
Choking, dyspnea	**0.678**	0.388	0.789	**0.819**		0.774
Sleep disorders	**0.757**		0.786	**0.554**	0.376	0.799
Loss of ability to eat	**0.704**	0.409	0.784	**0.739**	0.401	0.757
**Dom 6 – DEPENDENCE/LOSS OF CONTROL**			0.835			0.759
Dependence on care of others		**0.808**	0.819		**0.853**	0.649
Loss of control over oneself	0.324	**0.774**	0.775		**0.761**	0.746
Decreased mental abilities	0.435	**0.678**	0.803	0.316	**0.445**	0.675
Being a burden to others		**0.836**	0.770		**0.910**	0.730
	51.1%	13.2%		50.8%	15.9%	

^a^Varimax with Kaiser Normalization, Coefficients below 0.3 suppressed, ^b^Cronbach’s alpha for domain, ^c^Cronbach’s alpha if item deleted

**Table 5:** Item-Total Correlation of APND-EoLC Domains

	Domains	Patients			Family		
		Mean	SD	Item-Total	Mean	SD	Item-total
Dom 1	End-of life control	75.5	17.5	0.322–0.482	67.3	15.9	0.269–0.477
Dom 2	Keeping a patient alive	59.3	17.5	0.313–0.568	68.8	14.9	0.220–0.498
Dom 3	Trust in doctor/treatment	69.4	24.4	0.661–0.724	78.8	20.5	0.559–0.709
Dom 4	Trust in social support	50.8	21.4	0.426–0.495	76.7	80.0	0.467–0.545
Dom 5	One’s own suffering	67.1	22.2	0.562–0.664	75.6	18.4	0.454–0.710
Dom 6	Dependence/loss of control	79.3	21.4	0.604–0.721	79.2	21.4	0.475–0.654

**Fig. 2:**
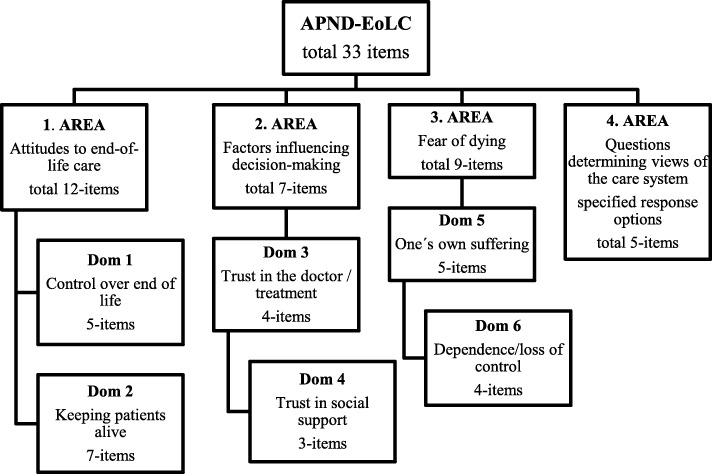
Structure of the APND-EoLC questionnaire

After the exploratory factor analysis all proposed items (*n* = 33) were left in the questionnaire. The structure of the created questionnaire is shown in Fig. 2.

### Correlation analysis

#### Item-Total correlation

The suggested minimum standard for item-to-scale total consistency is a correlation of at least 0.40 between the scale and each item. The correlation coefficient r < 0.40 was found in domain 1 in the item *“More open discussions in public about death and dying”* (patients: r = 0.322, family: r = 0.269) and in domain 2 in the item *“Discussion with a doctor about prognosis and end of life is too depressing”* (patients: r = 0.313, family: r = 0.220), see Table 5.

Correlation analysis showed that only some domains were highly inter-correlated (Table 6).

**Table 6:** Inter-Domain Correlations (Spearman) of APND-EoLC

	PATIENTS (P)						FAMILY MEMBERS (FM)					
P	Dom1	Dom2	Dom3	Dom4	Dom5	Dom6	Dom1	Dom2	Dom3	Dom4	Dom5	Dom6
Dom1	1.000	−0.099	−0.087	−0.167*	0.205**	0.314**	0.436**	0.028	0.028	0.106	0.098	0.314**
Dom2	−0.099	1.000	0.484**	0.299**	0.188**	0.020	−0.004	0.442**	0.185	0.138	0.160	0.020
Dom3	− 0.087	0.484**	1.000	0.512**	0.278**	0.194**	0.028	−0.027	0.306**	0.164	0.177	0.194*
Dom4	0.167*	0.299**	0.512**	1.000	0.181*	−0.015	−0.075	0.076	0.175	0.074	0.079	−0,015
Dom5	0.205**	0.188**	0.278**	0.181*	1.000	0.598**	0.098	−0.080	0.178	0.430**	0.449**	0.598**
Dom6	0.314**	0.020	0.194**	−0.015	0.598**	1.000	0.197	−0.040	0.246**	0.321**	0.356**	0.948**
**FM**												
Dom1	0.436**	−0.004	0.028	−0.075	0.098	0.197	1.000	−0.030	−0.040	0.181	0.174	0.197
Dom2	0.028	0.442**	−0.027	0.076	−0.080	−0.040	− 0.030	1.000	0.216*	0.149	0.111	−0.040
Dom3	0.028	0.185	0.306**	0.175	0.178	0.246**	−0.040	0.216*	1.000	0.319**	0.332**	0.246**
Dom4	0.106	0.138	0.164	0.074	0.430**	0.321**	0.181	0.149	0.319**	1.000	0.961**	0.321**
Dom5	0.098	0.160	0.177	0.079	0.449**	0.356**	0.174	0.111	0.332**	0.961**	1.000	0.356**
Dom6	0.314**	0.020	0.194*	−0,015	0.598**	0.948**	0.197	−0.040	0.246**	0.321**	0.356**	1.000

*p < 0.05, **p < 0.001

## Discussion

In recent decades, research into end–of-life care of patients with progressive neurological disease has flourished. However, in a review of the literature, no evaluation scales identifying views of PND patients on end-of-life issues were found. For this reason, we developed a tool in order to evaluate attitudes towards ethical aspects of end-of-life care for patients with PND, and their family members. The main finding of this study was to demonstrate the adequate psychometric characteristics of the Attitudes of Patients with PND to End-of-Life Care questionnaire in the population of patients with PNDs, and their family members. Determining opinions on ethical issues to end-of-life care can help physicians, patients, and family members to develop individual care plans. Due to frequent cognitive, emotional and behavioral disorders in patients with PND [6, 39], it is advisable to establish these views in the first stages of the disease. For this reason, patients with a selected disease were included in the group 1 year after diagnosis.

Based on the individual interviews and the focus group discussion, four major areas of interest were identified: namely patient and family attitudes towards end-of-life care, factors influencing decisions about life-sustaining treatment, concerns and fears of dying, and opinions on the care system. Scale questions were not used in the last area. For this reason, the psychometric properties were evaluated only in the first three areas, where satisfactory reliability of all scales was established. In the first area of the end-of-life attitudes survey, we created two domains using factor analysis: end-of-life control and keeping patients alive. The option of being able to decide on treatment preferences is cited by Steinhauser et al. [40] in his study as an important area of care at the end-of-life, both from the point of view of patients and their family members, and from doctors and other health care professionals (nurses, social workers, chaplains). Control over the end of life is associated with an important principle of current medical ethics: the principle of autonomy [41].

The right to autonomy implies that a patient, when she/he is competent, has the right to exercise her/his preference for or refusal of treatment, except in circumstances, (for example, contagious disease) in which refusal may cause harm to others. This can be regarded as respect for contemporaneous choice (or contemporary autonomy). Competent patients may also want to make a choice about future contingencies, including those in which they will not be able to decide for themselves e.g., when they lapse into incompetency [42]. Collating end-of-life treatment options makes it possible to pre-formulate the wishes of patients and their family. Using the APND-EoLC questionnaire in clinical practice can help doctors “open” these topics with patients and family members.

However, Haškovcová [43] points out that the patients’ authoritative and unlimited autonomy cannot always be exercised in the process of dying. She understands the right to self-determination as a human right, which is mainly related to the dignity and freedom of a human being. However, no right can be exercised recklessly and without correlations. The exaggerated emphasis on the patient’s autonomous behavior negates the fact that the patient makes their wishes and judgments under the weight of their difficult situation. Some authors point to other ethical approaches, such as the ethics of care [44]. The ethics of care usually works with a conception of persons as relational, rather than as the self-sufficient independent individuals of the dominant moral theories [44]. It is a philosophical perspective that focuses on the unique ethical dimension of close relationships, emphasizes relationships over rules and principles [45]. The ethics of care emphasizes the vulnerability and interdependence of human beings. It concludes that ethics should not only deal with decision-making, but also the quality of relationships, e.g. in terms of continuity, openness, trust and reliability [44]. Open communication with the patient, health care professionals and family members about the end of life care can also be based on a mutual relationship.

If caregivers listen to the narratives of identity of patients, and engage in a deliberative dialogue they will better be able to attune their care to the needs of patients [46, 47]. Narrative gives a peculiar insight into the reality of others’ lives that enhances our understanding. Baldwin [48] talks about a narratively informed decision-making. Based on a systematic review, Winterbottom [49] points out that narrative information influenced decision making more than the provision of no additional information and/or statistically based information in approximately a third of the studies.

In the second area, we identified factors that can influence decision-making at the end of life. Two domains were identified: trust in a doctor/treatment (including questions about doctors’ recommendations, hope of better quality of life, and prolongation of life), and social support (including questions aimed at surrounding support - family, other patients, or media). How the support of doctors, health professionals or loved ones can influence the decision-making process is being established in this domain. Bergum and Dossetro [50] talk about so-called Relational ethics. The basic premise of relational ethics is that ethical decisions/actions are made within the context of a relationship. The fundamental nature of relational ethics is that ethical commitment, agency, and responsibility for self and to the other arises out of concrete situations which invariably involve relations between two or more people and affect two or more people [51].

The decision not to provide life-saving medical treatment is a complex, emotionally-charged and contentious issue for the patient, medical team, and family alike [52]. Evidence shows that, too often, patients’ wishes about their medical treatment at the end of life are not known by their doctors and/or families [53]. One reason for this is that our culture is predominantly ‘death denying’ [54, 55]. Discussing death and end-of-life issues may be uncomfortable, and is sometimes taboo. It is recommended that the physician responsible for overall patient care and treatment initiate a discussion about care planning shortly after diagnosing a life-threatening disease or condition. According to Detering et al. [53], and Teno et al. [56], evidence shows that providing good end-of-life care leads to better quality-of-life for patients before death.

In the third area, we identified concerns and fears of dying. We selected two domains, fear of own suffering, and fear of dependence and control over one’s life. Dealing with fear of dying is an important part of understanding patients and their family members in the context of their end-of-life care experience. A scale of assessment for fear of death and dying was created by Collett and Lester as early as 1969 [57]. This 28-item scale (The Collett-Lester Fear of Death Scale - CL-FODS) has been validated in several cultural contexts, evidencing acceptable psychometric characteristics [58–62]. It is mainly used to assess fear of death and dying among healthcare workers in a training context. Fear of dying was identified in patients with acute coronary syndrome [63, 64], and in the elderly [65]. Our questionnaire dealt only with fear of dying, not of death itself. The areas of fear of dying were formulated in nine items, for instance loneliness, dependence, loss of control, burden to others. These areas may be related to spirituality [66], so we recommend finding out religion or spirituality when using the questionnaire.

The fourth area consisted of questions about the place of end-of-life care, and about initiating discussion with a doctor about the end of life and with those who should decide on end-of-life care. In particular, finding the most appropriate place of care is currently a common research subject in different patient groups [31, 67–69], and patients with PND and their family members should not be overlooked.

The APND-EoLC questionnaire can be used in acute and chronic care facilities. It is recommended that it be administered to patients and family members it shortly after diagnosis and then whenever a medical condition deteriorates. The questionnaire should be administered by a nurse. Other healthcare professionals should have access to the questionnaire in the medical records, mainly because of communication about the future care plan with the patient and their family. In social service facilities, the questionnaire should be the basis for the creation of an individual care plan. In social service facilities, planning of the end of life care is regarded as an extension of the individual plan, which supports the autonomy of clients of residential facilities. When planning, clients are asked about ideas and wishes regarding health care, as well as further self-care at the end of their lives [70]. The APND-EoLC questionnaire covers all these areas. It can also be administered by a nurse in social service facilities.

### The limits of the study and future research

In the first phase of creating a questionnaire, a certain limitation of the study is the low participation rate of patients and family members in focus groups. The reason was the patients’ and family members’ unwillingness to attend a group meeting. For this reason, we conducted individual interviews. On their basis we analyzed potential end-of-life issues from the perspective of patients and family members and presented them to the focus group participants. This could take into account the patients’ and family members’ perspective when developing the questionnaire. Another limitation of the creation of the questionnaire can be the selection of only two patients and two family members who assessed the understandability of the final version of the questionnaire. However, both patients and family members confirmed the clarity of the questions presented during the interview. Experts or target-population can evaluate the created items. Experts had evaluated individual items in our research earlier than patients and family members (target-population) did. According to Boateng et al. [71] expert judges seem to be used more often than target-population judges in scale development work to date. Ideally, one should combine expert and target population judgment, as was the case in our research (though resources of target-population were constrained).

The limitation of the evaluation of the psychometric properties of the created version of the questionnaire is that we do not use any comparator gold standard instrument. The reason is the lack of a validated tool in Czech for evaluating attitudes to ethical aspects of the end of life. In further research, the Collett-Lester Fear of Death Scale - CL-FODS could be used for area 3. For further research, we also recommend evaluating the tool over time for evidence of test-retest reliability and responsiveness to change. We also recommend testing a confirmatory factor analysis on the next sample of respondents.

## Conclusion

In conclusion, the Czech version of the APND-EoLC questionnaire had good psychometric characteristics when applied to patients with PND and their family members, and can be recommended for use in research and clinical assessment in the Czech Republic. The use of instruments with weak psychometric properties can seriously compromise the credibility of research findings. An English language version has been created for use in other countries. Able to be administered by doctors, nurses and social workers, the APND-EoLC questionnaire could become a suitable tool for determining end of life care plans.

## Supplementary information

**Additional file 1 **: questionnaire. The APND-EoLC - *The Attitudes of Patients with Progressive Neurological Disease to End-of- Life Care Questionnaire.*

## Data Availability

The excel file can be provided on demand. RB (corresponding author) should be contacted by anyone requesting the data.

## Abbreviations

AEOLI: Attitudes of Older People to End-of-Life Issues
APND-EoLC: The Attitudes of Patients with Progressive Neurological Disease to End-of-Life Care questionnaire
CL-FODS: The Collett-Lester Fear of Death Scale
EoLC: End of life care
EAN: The European Academy of Neurology
HD: Huntington’s disease
KMO: The Kaiser-Meyer-Olkin
MMSE: Mini-Mental State Examination
\MND: Motor neuron disease
MS: Multiple sclerosis
PD: Parkinson’s disease
PND: Progressive neurological diseases
