# Supplementary material for: Development and psychometric evaluation of a new tool for measuring the attitudes of patients with progressive neurological diseases to ethical aspects of end-of-life care
Source: BMC Med Ethics. 2020 Apr 15;21:28. doi: 10.1186/s12910-020-00471-9 (PMC7161107; doi:10.1186/s12910-020-00471-9)
Supplement: Supplementary file 1 — Additional file 1 : questionnaire. The APND-EoLC - The Attitudes of Patients with Progressive Neurological Disease to End-of- Life Care Questionnaire. [file 12910_2020_471_MOESM1_ESM.docx]

**The APND-EoLC**

The Attitudes of Patients with Progressive Neurological Disease to End-of- Life Care Questionnaire

version for patients (notes for family members version - FM)

1. **I would like a doctor to talk to me (FM: my loved one) openly about the prognosis and severity of my (FM: his/her) disease:**
2. at the start of the disease
3. in the case of deterioration of health
4. if I ask him/her myself (FM: if my loved one asks them)
5. not at all
6. other, specify:
7. **During interview with a doctor about the severity and prognosis of my (FM: his/her) disease, I would like to:**
8. be alone (FM: my loved one be alone)
9. be accompanied by a family member (FM: be with my loved one)
10. have a psychologist´s support (FM: my loved one to have a ….)
11. have a hospital chaplain´s support (FM: my loved one to have a ….)
12. other, specify:
13. **I would like my family to have all the information about the severity of my disease right from the start of the disease** (FM: I would like to know all the information about the severity of his/her illness right from the start of the disease if my loved one agrees):

1 2 3 4 5 6 7 8 9 10

strongly disagree strongly agree

1. **Discussion with a doctor about prognosis and end of life is too depressing.**

1 2 3 4 5 6 7 8 9 10

strongly disagree strongly agree

**NOW IMAGINE THAT YOUR DISEASE (FM: YOUR LOVED ONE´S DISEASE) HAS PROGRESSED SIGNIFICANTLY, AND DOCTORS, BASED ON EXPERIENCE, ASSUME THAT YOUR (FM: HIS/HER) LIFE SPAN WILL NOT EXCEED 3-6 MONTHS.**

1. **In this situation, I would like to have the latest treatment (FM: for my loved one) available regardless of the side effects.**

1 2 3 4 5 6 7 8 9 10

strongly disagree strongly agree

1. **In this situation, the doctor should decide about and manage end-of-life care (FM: of my loved one).**

1 2 3 4 5 6 7 8 9 10

strongly disagree strongly agree

1. **In this situation, I would like to (FM: my loved one to be):**
2. be at home
3. be in the hospital
4. be in the hospice
5. be in the social facility (residential institution)
6. be in the long-term care facility (nursing home)
7. **In this situation, I would like to have medicine available to end my life (FM: his/her life).**

1 2 3 4 5 6 7 8 9 10

strongly disagree strongly agree

1. **In this situation, quality of my life (FM: his/her life) would be more important for me than length of life.**

1 2 3 4 5 6 7 8 9 10

strongly disagree strongly agree

1. **In this situation, I have greater fear of helplessness and dependence (FM: of my loved one) than of death (FM: his/her death).**

1 2 3 4 5 6 7 8 9 10

strongly disagree strongly agree

1. **In this situation, I would like to have pain relief treatment (FM: for my loved one) at the cost of sedation or confusion.**

1 2 3 4 5 6 7 8 9 10

strongly disagree strongly agree

1. **In this situation, I would like to have pain killers under control. I wouldn't like to rely on my nurse or doctor to give them to me (FM: to my loved one).**

1 2 3 4 5 6 7 8 9 10

strongly disagree strongly agree

1. **In this situation, if I (FM: she/he) lost the ability to eat, I would like to start tube (enteral) feeding.**

1 2 3 4 5 6 7 8 9 10

strongly disagree strongly agree

1. **In this situation, if I (FM: she/he) lost the ability to breathe, I would like to introduce APV.**

1 2 3 4 5 6 7 8 9 10

strongly disagree strongly agree

1. **In this situation, I would like to be kept alive at any cost (FM: my loved one to be kept..).**

1 2 3 4 5 6 7 8 9 10

strongly disagree strongly agree

**IF YOU WERE CONSIDERING A DECISION TO INTRODUCE ARTIFICIAL NUTRITION OR MECHANICAL VENTILATION (FM: for your loved one), HOW MUCH THE FOLLOWING SITUATIONS WOULD AFFECT YOUR DECISION:**

NOT AT ALL VERY MUCH

1. Doctor’s recommendation 1 2 3 4 5 6 7 8 9 10
2. More doctors’ consensus 1 2 3 4 5 6 7 8 9 10
3. Hope of a better quality of life 1 2 3 4 5 6 7 8 9 10
4. Hope of prolonging life 1 2 3 4 5 6 7 8 9 10
5. The approval of the closest family 1 2 3 4 5 6 7 8 9 10
6. Information from mass media and the Internet 1 2 3 4 5 6 7 8 9 10
7. Other patients’ experiences 1 2 3 4 5 6 7 8 9 10
8. **If it was necessary to make a decision whether to keep me (FM: my loved one) alive on life support or not, who should make this decision:**
9. a doctor
10. a medical consilium
11. a family member designated by the family
12. a family member of my own choice in advance (FM: a family member designated by the patient)
13. I would like to make my own decision in advance that doctors would respect (FM: my loved one would make his/her own decision in advance …)
14. only FM: I would like to decide about it regardless of opinion of my loved one or other family members´

**HOW MUCH ARE YOU WORRIED ABOUT THE FOLLOWING SITUATIONS WHEN THINKING ABOUT YOUR DYING (FM: HIS/HER DYING)**

NOT AT ALL VERY MUCH

1. Severe pain 1 2 3 4 5 6 7 8 9 10
2. Loneliness 1 2 3 4 5 6 7 8 9 10
3. Choking, dyspnea 1 2 3 4 5 6 7 8 9 10
4. Sleep disorders 1 2 3 4 5 6 7 8 9 10
5. Loss of ability to eat 1 2 3 4 5 6 7 8 9 10
6. Dependence on care of others 1 2 3 4 5 6 7 8 9 10
7. Loss of control over oneself 1 2 3 4 5 6 7 8 9 10
8. Decreased mental abilities 1 2 3 4 5 6 7 8 9 10
9. Being a burden to others 1 2 3 4 5 6 7 8 9 10
10. **I would like more open discussions in public about death and dying.**

1 2 3 4 5 6 7 8 9 10

strongly disagree strongly agree
